# Monte Carlo Simulation and Experimental Studies of CO_2_, CH_4_ and Their Mixture Capture in Porous Carbons

**DOI:** 10.3390/molecules26092413

**Published:** 2021-04-21

**Authors:** Pakamas Kohmuean, Worapoj Inthomya, Atichat Wongkoblap, Chaiyot Tangsathitkulchai

**Affiliations:** School of Chemical Engineering, Suranaree University of Technology, Nakhon Ratchasima 30000, Thailand; m6112479@g.sut.ac.th (P.K.); worapojinthomya@gmail.com (W.I.); chaiyot@sut.ac.th (C.T.)

**Keywords:** activated carbon, adsorption, carbon nanotube, Monte Carlo simulation, CO_2_, CH_4_

## Abstract

Adsorption of carbon dioxide and methane in porous activated carbon and carbon nanotube was studied experimentally and by Grand Canonical Monte Carlo (GCMC) simulation. A gravimetric analyzer was used to obtain the experimental data, while in the simulation we used graphitic slit pores of various pore size to model activated carbon and a bundle of graphitic cylinders arranged hexagonally to model carbon nanotube. Carbon dioxide was modeled as a 3-center-Lennard-Jones (LJ) molecule with three fixed partial charges, while methane was modeled as a single LJ molecule. We have shown that the behavior of adsorption for both activated carbon and carbon nanotube is sensitive to pore width and the crossing of isotherms is observed because of the molecular packing, which favors commensurate packing for some pore sizes. Using the adsorption data of pure methane or carbon dioxide on activated carbon, we derived its pore size distribution (PSD), which was found to be in good agreement with the PSD obtained from the analysis of nitrogen adsorption data at 77 K. This derived PSD was used to describe isotherms at other temperatures as well as isotherms of mixture of carbon dioxide and methane in activated carbon and carbon nanotube at 273 and 300 K. Good agreement between the computed and experimental isotherm data was observed, thus justifying the use of a simple adsorption model.

## 1. Introduction

Adsorption of carbon dioxide and methane has been of significant interest to adsorption science and engineering because carbon dioxide is a potential molecular probe for characterizing narrow micropores at experimentally measurable pressures [1] while the study of methane adsorption helps to synthesize potential porous solid absorbents for methane storage in transportation vehicles as well as the promotion of researches in fuel cell technology [2,3]. In addition, carbon dioxide and methane are implicated as greenhouse gases that can cause global warming due to greenhouse effects; therefore, the reduction of greenhouse gases has become increasingly important [4,5,6]. The adsorption process is one of the technologies used to control CO_2_ emission to atmospheric environment by the removal of CO_2_ from gas mixtures [7,8]. Furthermore, mixtures of CO_2_ and CH_4_ are major constituents in fermented-biogas while mixtures of CO_2_ and hydrocarbons are normally found in natural gas. The removal of CO_2_ from these gas mixtures is of utmost importance for in enhanced energy utilization [9,10]. Adsorption techniques such as temperature-swing and pressure-swing adsorption together with using porous adsorbents, especially porous carbons, are among the most promising candidates for these carbon dioxide capture [7]. Therefore, the characterization of porous carbon (for example, pore size distribution (PSD)) is necessary for the utilization and design of promising porous solids for a variety of applications. However, the evaluation of activated carbons for a given separation is still a difficult task; this is due to the difficulty in predicting their adsorption behaviors for a suitable model representing the structure of carbon [7].

On the other hand, agricultural production can generate a huge amount of agricultural waste and biomass such as coconut shells, corn cops and longan seeds, and it translates into a great impact on the environment [3,11]. Therefore, the recycling process and utilization of such abundant waste materials are an urgent issue nowadays. However, the collection, processing and transportation costs of those waste materials are higher than their economic value for beneficial use [11]. The longan fruit is one of the most valuable consumable fruits and is widely growth in Thailand. Its seeds are considered as agricultural waste produced from many processing plants. The longan seeds have a fixed carbon content of 19.6% with relatively low ash content and high volatile content [12]. The fixed carbon composition of longan seed compared with other carbonaceous materials can promote its use as a precursor for activated carbon synthesis. Activated carbon is one of the most widely used commercial adsorbents in the area of in adsorption because of its large micropore and mesopore volumes and high surface area, while single wall carbon nanotube (SWCN) has been increasingly used in a number of applications because of its strong fluid-solid interaction and electronic properties [13,14]. Therefore, porous carbons become the promising and sustainable alternative materials for energy storage such as natural gas (NG) and hydrogen [15,16]. Methane is the major composition contained in NG and can be stored in porous materials as an adsorbed phase called ANG. The maximum theoretical methane uptake and delivery for activated carbon at 3.5 MPa and 25 °C are 213 *v*/*v* and 120 *v*/*v*, respectively [17]. The activated carbon fibers, powered activated carbons and activated carbon monoliths have been found to be suitable materials for methane storage and the volumetric CH_4_ uptake and delivery for these materials were about 163 *v*/*v* and 143 *v*/*v*, respectively [15]. The methane storage capacity at 7 °C and 8.5 MPa on activated carbon with surface area of 1060 m^2^/g was 113.5 *v*/*v* [16]. The activated carbon derived from peanut shell has the adsorption capacities of CH_4_ at 1 bar and 40 °C of 1.38 mmol/g [3]. Therefore, the activated carbon derived from longan seed may be considered in this study.

Molecular simulations have been introduced to study the adsorption of fluids in nanoporous carbons and compared with the experimental data. Generally, activated carbon is modeled as a collection of pores of different widths. The pores are assumed to be either infinite carbon slit pores [1,18,19,20] or infinite carbon cylindrical pores [21,22]. However, the infinite pore model is too ideal to reflect the adsorption behavior of activated carbon whose length is finite [23] and it contains chemical impurities, functional groups and defects on the basal graphene layers [24]. While the isolated SWCN have been commonly used in many simulations, this simplistic model does not truly represent real carbon nanotubes because these tubes are usually found in tangled or partly aligned bundles due to the strong Van der Waals interactions [25,26]. Therefore, in this study, real carbon pores of finite length and carbon surfaces as graphene layers comprising carbon atoms arranged in a hexagonal pattern proposed in our previous studies [27,28] are used to model the structure of activated carbon for studying the adsorption behavior of carbon dioxide and methane in porous carbons. The detail of solid models are described in Section 2.2.

In this study, adsorption isotherms of CO_2_ and CH_4_ at 273 and 300 K were obtained for graphitic slit pores having widths of 6.3 to 30 Å by using a Grand Canonical Monte Carlo (GCMC) simulation, and they were used as a kernel to characterize the experimental data obtained for commercial activated carbon (CAC) derived from coconut shells and longan seed activated carbon (LAC). The homogeneous nanotubes bundles were also used to investigate the adsorption of CO_2_ and CH_4_ in carbon nanotubes by using the GCMC ensemble. The tube diameters of 9.5, 10.8, 13.6 and 16.3 Å were selected for investigation the effect of tube diameter on adsorption isotherm. The simulation results were applied to characterize the pore size distribution (PSD) of commercial single walled carbon nanotubes (SWCNs) and compared with the experimental data. The reconstructed adsorption isotherm obtained from the PSD of CO_2_ and CH_4_ in finite-length pores are used to predict the adsorption isotherm obtained for the binary mixtures and compare with the experimental data. In this paper, we investigate the abilities of Pore Size Distribution (PSD) obtained for finite-length pore model to predict pure and binary adsorption of carbon dioxide and methane in activated carbon and carbon nanotubes.

## 2. Methodology

### 2.1. Fluid-Fluid Potential Models

In this study, methane is modeled with a single Lennard-Jones (LJ) site while carbon dioxide is modeled as a 3-center LJ molecule with fixed partial charges to account for the quadrupole moment [29]. Nitrogen is represented as a non-spherical model with two LJ sites located at the center of nitrogen atoms separated by 1.1 Å, a positive charge (12.98 × 10^−20^ C) at the center of the molecule and two negative charges (−6.49 × 10^−20^ C) at the centers of the two nitrogen atoms [30,31]. The molecular parameters are listed in Table 1 for methane and nitrogen and Table 2 for carbon dioxide.

A cut-off radius in the calculation of interaction energy of five times the collision diameter of fluid (5$\sigma_{ff}$ or 5σ^C-C^ in the case of CO_2_) is used in this study.

### 2.2. Solid Models

A simple graphitic slit pore of finite length was used to model activated carbon [27]. The pore wall consists of three graphene layers with an interlayer spacing of 3.354 Å. Each graphene layer is composed of carbon atoms arranged in a hexagonal pattern with C-C bond length of 1.42 Å, and we chose square graphene layers with linear dimension of 60 Å [23] in our simulation. A range of pore width from 6.3 to 50 Å was used in the simulation of local isotherms for the subsequent derivation of pore size distribution of activated carbon. 

**Figure 1:**
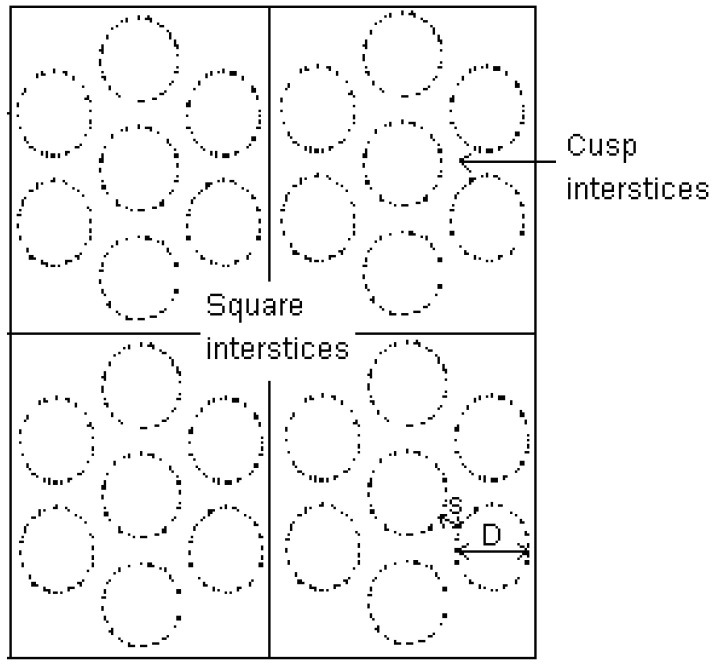
A schematic diagram of a cross section of bundles of single wall carbon nanotube (SWCN) and the minimum image convention used in this study.

Single wall carbon nanotube (SWCN) was modeled as an ensemble of bundles of seven graphitic cylinders arranged in a hexagonal pattern, as shown in Figure 1. Each cylinder has the following characteristics: (1) its diameter is defined as the diameter of a circle passing through the centers of carbon atoms, and (2) the C-C separation distances is 1.42 Å, which is the same as that in the flat graphene. The minimum separation spacing of carbon atoms on the external surface of two adjacent nanotubes is denoted as *s,* which is treated as a variable in this study (chosen between 4 and 10 Å). For this model of SWCN, two different types of interstices were identified: (i) the space between the individual nanotubes, i.e., the cusp interstices, and (ii) the space at the corner of simulation box which formed the square interstices. The cylindrical pores used in this study are (7:7), (8:8), (10:10) and (12:12) SWCNs, which correspond to the diameters of 9.5, 10.8, 13.6 and 16.3 Å, respectively [27].

The LJ parameters for a carbon atom in a graphene layer, σ_ss_ and ε_ss_/k, are 3.4 Å and 28 K, respectively [1]. The interaction energy between a site of a fluid molecule and a carbon atom on the graphene layer was calculated with the LJ 12-6 equation. The cross molecular parameters were calculated from the Lorentz-Berthelot rule [32].

### 2.3. Monte Carlo Simulation

We adopted the Metropolis algorithm in the Monte Carlo (MC) simulation [32] and the GCMC ensemble was used to obtain the adsorption isotherms. In the case of slit pore, the simulation box for this ensemble is a finite length carbon slit pore, and has a linear dimension of about 60 Å in the x and y directions. We assumed that the top and the bottom of the simulation box are two walls of the slit pore, and each wall consists of three graphene layers. We specified the volume of the box (i.e., pore volume), the chemical potential and the temperature of the system to obtain the adsorption equilibrium. One GCMC cycle consists of one thousand displacement moves and attempts of either insertion or deletion with equal probability. In each displacement move, the particle is also rotated randomly around x, y or z axis with equal probability in the case of nitrogen with 2 LJ centers and carbon dioxide with 3 LJ centers. For an adsorption branch of the isotherm 20,000 cycles for the calculation are typically needed for the system to reach equilibrium. For each point on the adsorption branch, we used an empty box as the initial configuration, and the simulation was carried out until the number of particles in the box does not change in statistical sense. The displacement step length was initially chosen as 0.5 times the collision diameter of fluid, and it is decreased by 5% when the acceptance ratio is less than 0.5 and increased by 5% when this ratio is greater than 0.5. In the finite length pores, the usual periodic boundary conditions are not applied. Instead, the reflecting boundary conditions are applied in x, y and z directions as described in the literature [33], where the particle move is rejected if the attempted displacement puts the selected particle outside the simulation box. This boundary condition is applied to prevent particles from going outside of the simulation box. The pressure of the bulk gas corresponding to a given chemical potential are calculated from the equation of state proposed by Johnson et al. [34]. This is because the densities of coexisting liquid and vapor phases and saturation pressures for bulk CO_2_ obtained from experimental data agreed well with the Gibbs ensemble Monte Carlo (MC) simulation for three-center model of Harris and Yung [29] and the MC simulation results calculated with equation of state of Johnson et al. [35]. However, the computation time is shorter in the case of Johnson et al.’s equation of state [35]. 

The simulation box for carbon nanotubes bundle is a rectangular box with the z-dimension being the same as the tube length, while the dimensions in the x- and y-directions depend on the tube diameter and spacing to produce a replica of seven tubes in the neighboring boxes. A tube length of 50 Å was chosen in this study. The diameters of SWCN (D) are 9.5, 10.8, 13.6 and 16.3 Å, and the separation spacing between tube walls is 4, 7 and 10 Å. The length in x and y directions can be calculated from these equations [28].

(1) $$L_{x}=3\left(D+s\right);\;L_{y}=2\left\{\left(\frac{3D}{2}+s\right)\times sin\left(\frac{\pi }{3}\right)\right\}+s$$

Similar to the slit pore model, the GCMC method is also used to obtain the adsorption isotherm of fluid in a bundle of seven tubes. However, in the case of tube bundle, periodic boundary conditions (PBCs) [32,33] are applied in *x* and *y* directions. The average pore density (*ρ_av_*) can be defined as the ratio of the number of particles inside the pores to the pore volume [28]:

(2) $$\rho_{av}=\frac{‹N_{inside}›}{V_{pore}}$$

where *V_pore_* = 7πR^2^L, where R is the subtraction of half a collision diameter of carbon atom from the pore radius, L is the pore length and *N_inside_* is the number of particles inside nanotubes. While the average outside density, volume is defined as L_x_L_y_L_z_ − (7π(R + σ_ss_)^2^L), where L_x_, L_y_ and L_z_ are linear dimensions of the box, and N_outside_ is defined as N-N_inside_, where N is total number of particles. In the case of slit pore, V_pore_ = L_x_L_y_(H − σ_ss_), where H is the pore width.

### 2.4. Experiments

The experimental isotherms of CO_2_ and CH_4_ at 273 and 300 K, and mixtures of CO_2_ and CH_4_ for various activated carbon and SWCN were obtained by using an Intelligent Gravimetric Analyzer (IGA) model IGA-002 supplied by Hiden Analytical Ltd. (Worrington, England). Prior to each adsorption run, the carbon sample (0.12 g) was outgassed at 200 °C for 10 h and then allowed to cool down to the adsorption temperature. Due to the limitation of our equipment (IGA), the same sample was used to measure the adsorption uptake obtained for single component in both mmol/g and mg. While the total adsorbed amount obtained for gas mixture was measured in mg, we cannot measure the adsorption uptake for each gas separately. Adsorbents used in this study are longan seed derived activated carbons (LAC): LAC1 and LAC2, and carbon nanotubes supplied by Chengdu Organic Chemicals, Chengdu, China. The porous properties of the derived activated carbons and carbon nanotube are measured by nitrogen adsorption at 77 K using an Accelerated Surface Area and Porosimetry Analyzer (ASAP2010, Micromeritics Instrument Corporation, Norcross, GA, USA). Brunauer-Emmet-Teller (BET) method was utilized to determine the specific surface area and micropore volumes calculated from the Dubinin-Astakhov equation. The Density Functional Theory (DFT) was applied to obtain the Pore Size Distribution (PSD).

## 3. Results and Discussions

We start our discussion with the experimental isotherm data for pure component and mixture. Next, we analyze the GCMC simulated isotherms to study the effects of curvature on adsorption. Using these simulated isotherms in the analysis of pure component isotherms we derived pore size distributions (PSD) of activated carbon and carbon nanotube. Finally, these PSDs were used to describe adsorption data of carbon dioxide and methane mixtures. 

### 3.1. Experimental Isotherms

**Figure 2:**
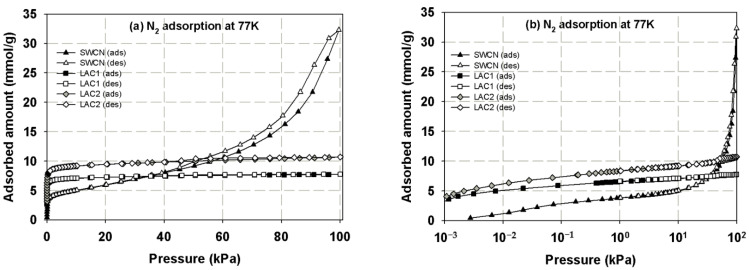
Adsorption isotherms of nitrogen at 77 K in various kinds of carbon (filled symbols for adsorption and unfilled symbols for desorption) in linear (**a**) and semi-log (**b**) scales.

**Figure 3:**
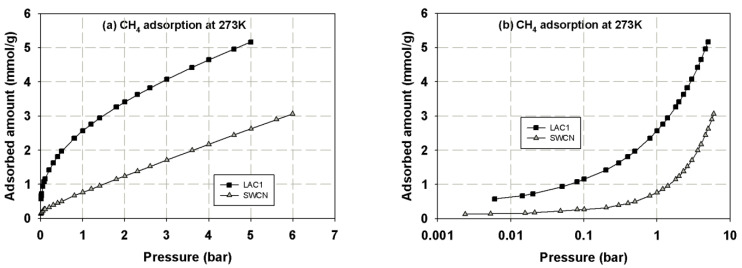
Adsorption isotherms of methane in activated carbons and single wall carbon nanotube at 273 K in linear (**a**) and semi-log (**b**) scales.

**Figure 4:**
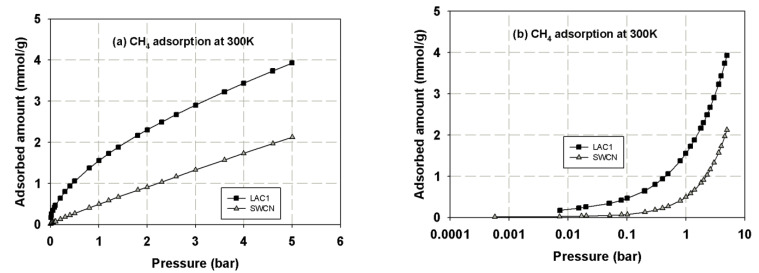
Adsorption isotherms of methane in activated carbons and single wall carbon nanotube at 300 K in linear (**a**) and semi-log (**b**) scales.

**Figure 5:**
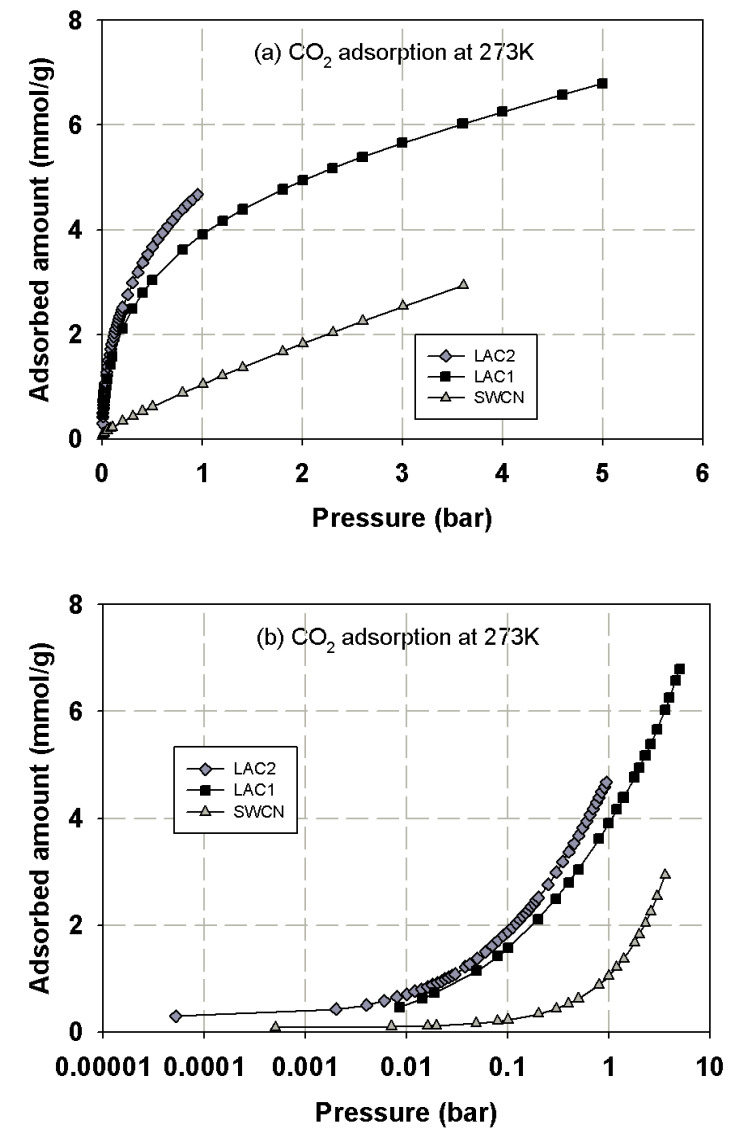
Adsorption isotherms of carbon dioxide at 273 K in various kinds of carbons in linear (**a**) and semi-log (**b**) scales.

**Figure 6:**
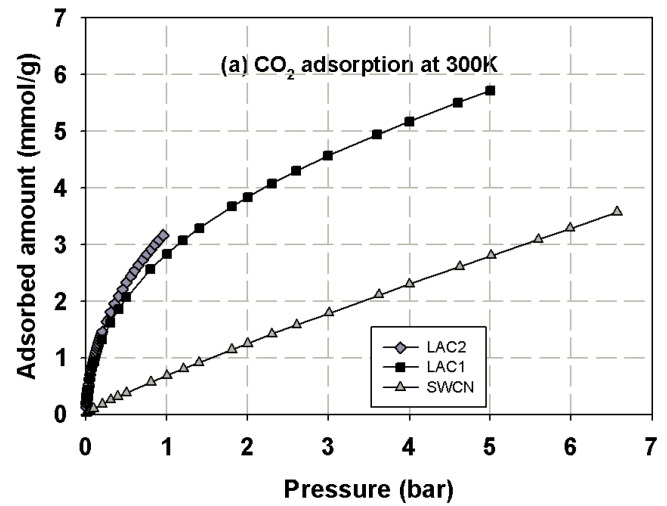

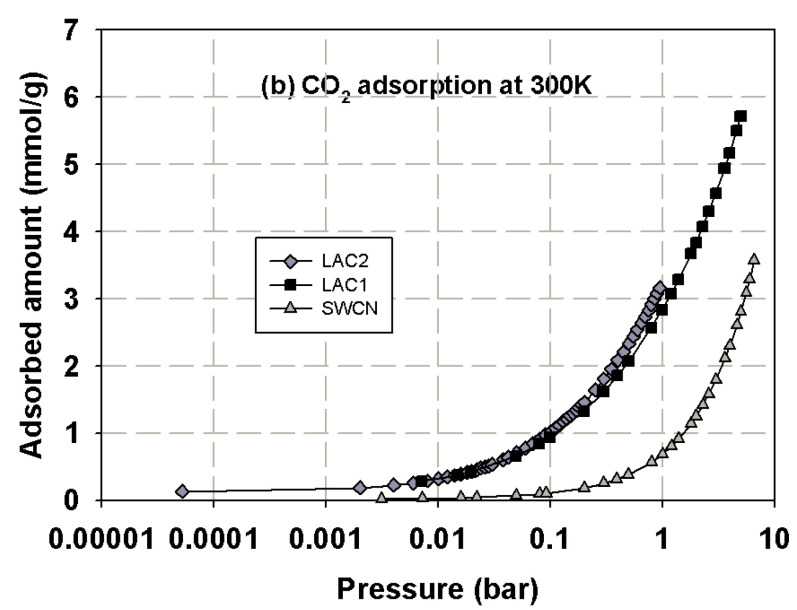
Adsorption isotherms of carbon dioxide at 300 K in various kinds of carbons in linear (**a**) and semi-log (**b**) scales.

**Figure 7:**
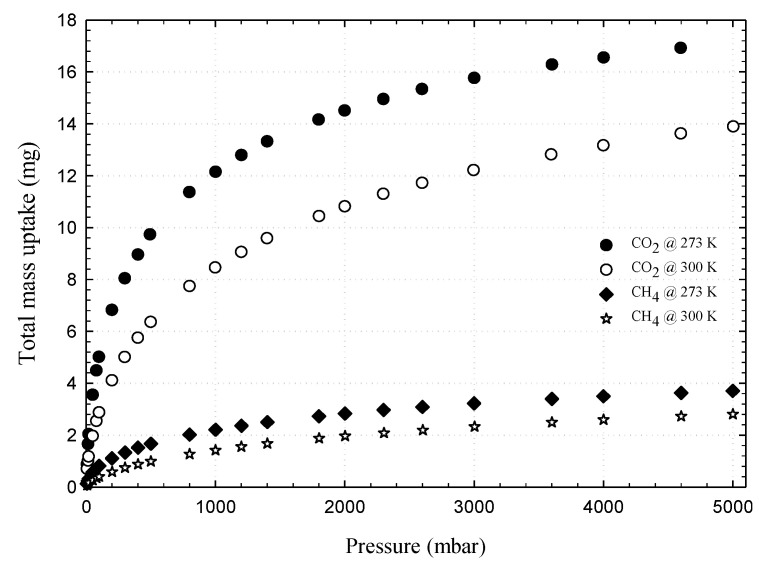
Experimental adsorption isotherms of CO_2_ and CH_4_ in LAC1 activated carbon at 273 and 300 K.

**Figure 8:**
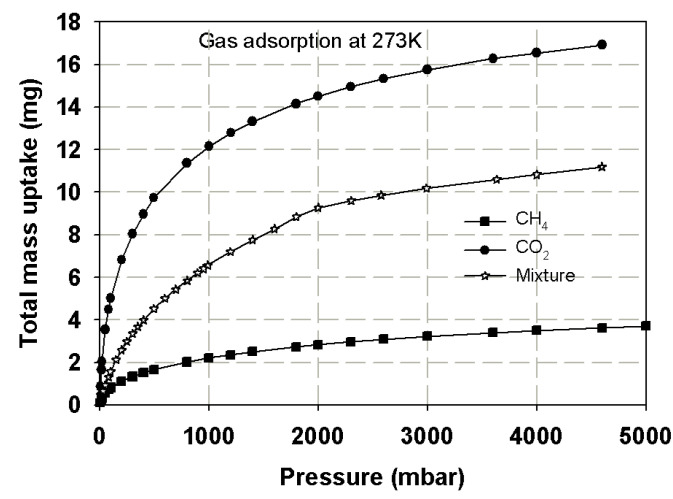
Adsorption isotherms of methane, carbon dioxide and their mixture in LAC1 activated carbon at 273 K.

First, we present the adsorption isotherms of nitrogen at 77 K obtained for activated carbons and single wall carbon nanotubes, as presented in Figure 2 and these isotherms are used to determine the porous properties of tested carbons such as BET surface area, micropore volume and total pore volume as shown in Table 3. The adsorption isotherms of methane and carbon dioxide presented as adsorbed amount in mmol/g against pressures at 273 and 300 K for various carbons are presented in Figure 3, Figure 4, Figure 5 and Figure 6, in linear (a) and semi-log (b) scales. We also plot the total mass uptake of both species in LAC1 against pressures at different temperatures as shown in Figure 7 and use these plots to compare with the adsorption of their mixture as shown in Figure 8.

The experimental isotherms for nitrogen adsorption in SWCN, LAC1 and LAC2 at 77 K versus pressure in linear (a) and semi-log (b) scales are shown as upward-triangle, square and diamond symbols, respectively, in Figure 2, and the filled symbols are those for adsorption and unfilled symbols are those for desorption. In the case of SWCN, the experimental data show a negligible adsorption at pressures lower 0.01 bar, and then increase gradually to about 0.2 bar, after which the slope of the isotherm increases steeply. It increases gradually due to the formation of monolayer inside and outside the tubes, and is followed by the dramatic increase in adsorption isotherm which is the result of multilayer adsorption outside the tubes [28]. Unlike SWCN, in the case of activated carbons, the experimental data show a continuous increase of adsorption with pressure at a low pressure range and approaches a constant value at higher pressures, indicating the type I adsorption isotherm. This type I isotherm indicates that the activated carbon used in this study is dominated by microporosity, and a small hysteresis loop is also observed. The porous properties of SWCN and activated carbons used in this study shown in Table 3 are derived by using the Density Functional Theory (DFT) and Dubinin-Astakhov equation which was supplied by Micrometrics together with the experimental data of nitrogen at 77 K. It was found that the specific surface area of SWCN is 853 m^2^/g and the total pore volume is 1.29 cm^3^/g, which are higher than LACs. The SWCN seems to be composed of mesopores and macropores, which is different from those of activated carbon, which contained mostly micropores.

The same behavior can be observed for carbon dioxide and methane; the experimental data show a continuous pore filling mechanism which is a typical isotherm observed for many micropore adsorbents. However, there is no sharp change of phase transition observed under this temperature conditions. It is noted that this adsorption behavior of the studied fluids occurs at pressure lower than the saturated pressure. The adsorption of CO_2_ and CH_4_ in activated carbon is greater than that in SWCN. This is due to the smaller average pore size of activated carbon, which leads to the stronger interaction between fluid and pore wall, hence giving the increase in the adsorbed amounts of CH_4_ and CO_2_ for activated carbons. Therefore, the pore size of porous carbon is one of the deciding factors to be considered for the adsorption of CH_4_ and CO_2_. The effects of pore size on adsorption isotherm of fluid are presented in the next section.

Figure 7 shows the experimental adsorption isotherms obtained from IGA for carbon dioxide and methane in Longan seed activated carbon (LAC1) at 273 and 300 K. The adsorbed amount is presented in mass uptake (mg) as a function of pressure that varied from 5 mbar to 5000 mbar (0.5 MPa). For the adsorption of carbon dioxide, the mass uptake curves for the adsorption at 273 and 300 K and the pressure up to 5000 mbar resemble the initial part of the Type I isotherm, thus indicating the adsorption by micropore filling in LAC1 activated carbon [24,36,37,38]. It is further noted that the adsorbed amount at 273 K is higher than 300 K, showing the characteristic of physical adsorption with the liberation of heat and that the adsorbed molecules acquire the greater energy to evaporate [24,39]. For methane adsorption, the behavior of these isotherms is similar to carbon dioxide but their mass uptakes are much less because of the different in molecular structure. Methane molecules do not possess quadrupole moment from electrical charge as do the carbon dioxide molecules [29]. Therefore, the intermolecular force between solid surface and fluid molecule of methane are weaker than those of carbon dioxide, resulting in the differences of adsorption behavior of these two gases [40,41].

It has been reported in the literature that the adsorbed amount of methane in the carbon fiber obtained from petroleum pitch activated with CO_2_ and 73% burn-off degree (having surface area of 2400 m^2^/g) were 163 *v*/*v* and 143 *v*/*v* for CH_4_ uptake and delivered at 25° and 3.5 MPa), respectively [15]. The volumetric methane uptake and delivery values for the activated carbon fibers prepared from a commercial carbon fiber activated with CO_2_ and 74% burn-off degree (having surface area of 2862 m^2^/g) were 166 *v*/*v* and 150 *v*/*v*., respectively [15]. The storage capacities of CO_2_ and CH_4_ on activated carbons derived from agricultural wastes such as peanut shell were 3.63 and 1.38 mmol/g, respectively, while those derived from metasequoia leaf were 2.26 and 0.89 mmol/g, respectively, at 1 bar and 40 °C [3]. In this study, the activated carbons derived from longan seed, LAC1 and LAC2, have the CO_2_ capture capacities of 4.0 and 4.7 mmol/g at 1 bar and 273 K (0 °C), respectively, and those of 2.9 and 3.2 mmol/g at 1 bar and 300 K (27 °C). The CH_4_ storage capacities of LAC1 at 1 bar were 2.6 and 1.7 mmol/g at temperature of 273 and 300 K, respectively. When pressure increased to 5 bar (0.5 MPa), the CH_4_ capacities on LAC1 increased to 5.2 and 3.9 mmol/g at 273 and 300 K, respectively. The surface area obtained for AC derived from peanut shell and metasequoia leaf were 790 and 496 m^2^/g, respectively [3], while those of LAC1 and LAC2 were 538 and 705 m^2^/g, respectively. As one can see, the activated carbons derived from agricultural waste have less surface area compared with those obtained from petroleum pitch and commercial carbons; therefore, the surface area and micropore size distribution are the key factors to prepare the carbon materials for energy storage [15,16]. Preparation of activated carbons derived from agricultural wastes to achieve the US Department of Energy (US DOE) standard for methane storage will be investigated in our next study. 

**Figure 9:**
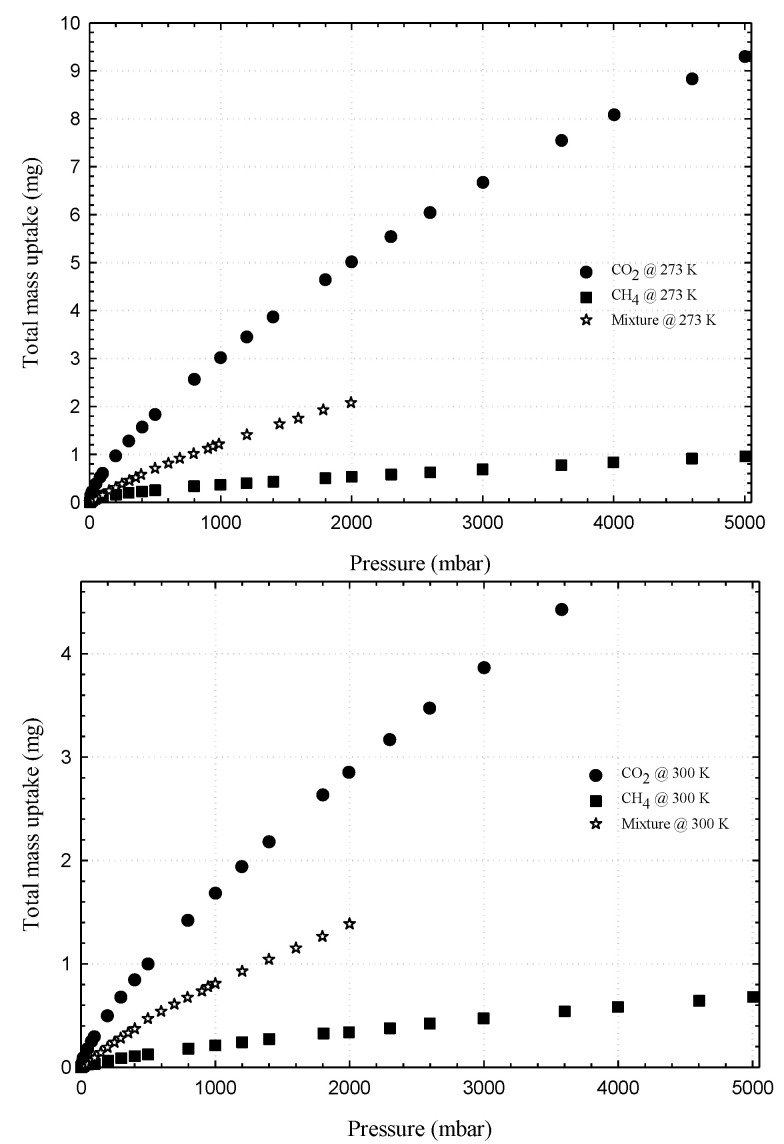
Adsorption isotherms of methane, carbon dioxide and their mixture in SWCN at 273 and 300 K.

Coadsorption equilibria of CO_2_/CH_4_ gas mixture with equi-molar feed composition on LAC1 and SWCN were measured at temperature of 273, and the results are presented in Figure 8 and Figure 9, respectively. These figures show the plot between total mass adsorbed of mixture and pressure; for comparison, the adsorption isotherm of pure components are also plotted in the same figure. In the case of gas mixture adsorption, the isotherm takes the same shape as that of the pure component adsorption and lies between the pure component adsorption of carbon dioxide and methane as shown in the figure. Due to the limitation of our instrument, the adsorption uptake of each component could not be separately measured; thus, only the total mass adsorbed of the mixed gases could be registered. However, the isotherm of gas mixture does indicate that carbon dioxide is more strongly adsorbed than methane due to its higher boiling point and the possession of a quadrupole moment [7,42,43]. It has been reported in the literature that, if concentrations of this binary gas mixture are varied, the total adsorbed amount will decrease with a decreasing of carbon dioxide concentration in the mixture [42,43,44,45]. CO_2_ and CO_2_/CH_4_ mixture adsorption in SWCN at high pressures behave as Langmuir isotherm, and this is due to the formation of monolayer inside the tubes [37]. In Figure 9, the isotherm of carbon dioxide, methane and their binary mixture adsorption tend to show similar behavior; the adsorbed amount at 273 K is larger than 300 K from reason of exothermic process as described in previous section.

### 3.2. Simulation Isotherm for Gas Adsorption in Finite-Length Slit Pore

**Figure 10:**
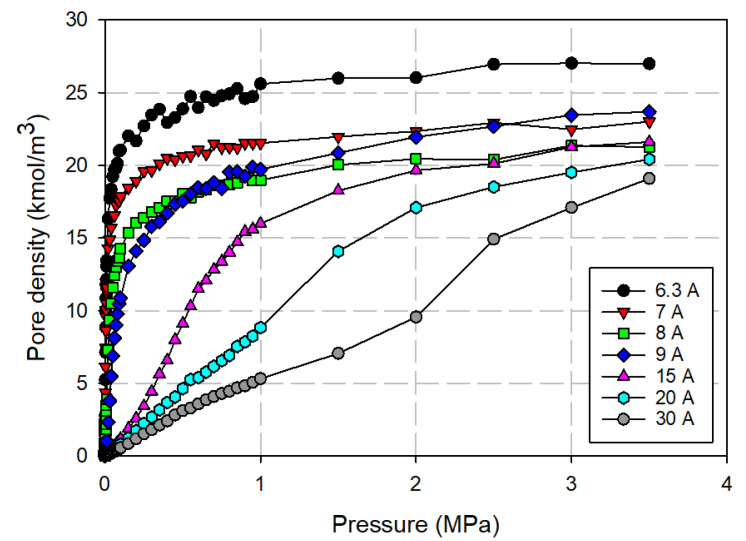
Adsorption isotherms of carbon dioxide obtained for a finite-length slit pore at 273 K.

**Figure 11:**
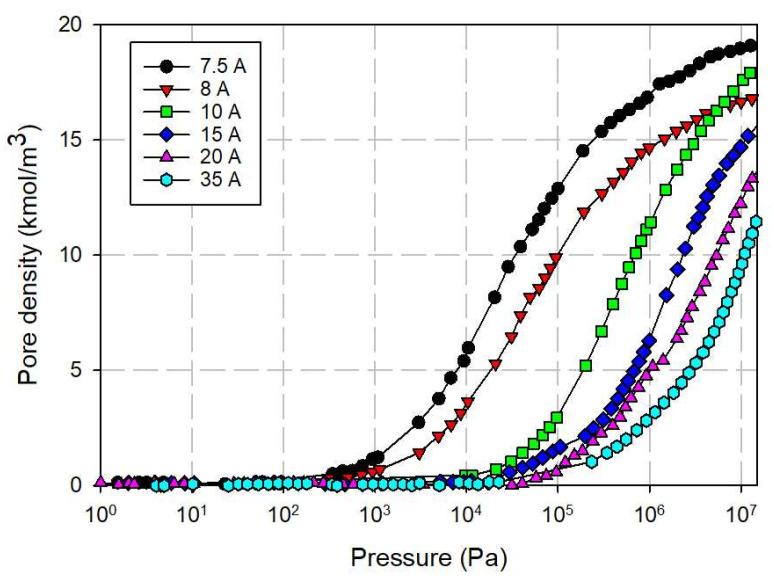
Adsorption isotherms of methane obtained for a finite-length slit pore at 273 K.

**Figure 12:**
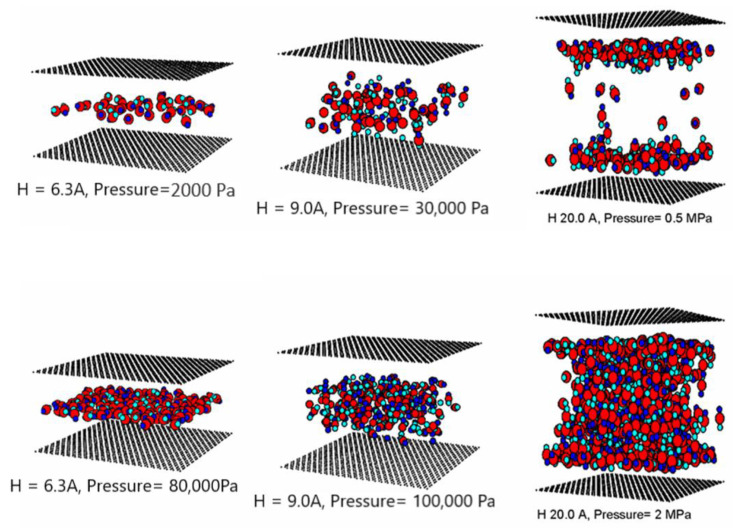
Snapshots of CO_2_ in 6.3, 9 and 20 Å pores at 273 K.

The simulated isotherms versus pressures for pure CO_2_ and CH_4_ at 273 K in single carbon slit pores of various pore widths (from 6.3 to 40 Å) are obtained by using the GCMC method; for clarity, only selected isotherms are shown in Figure 10 and Figure 11, respectively. The snapshots of pure CO_2_ molecules inside the pores at various pressures are also shown in Figure 12 to study the adsorption behaviour of gas in different pore widths.

The simulation isotherms of pure CO_2_ and CH_4_ in the slit pore model show the continuous pore filling of a single layer for the width less than 9.5 Å. The isotherm drops with increasing of pore widths. This is due to the weaker interaction between fluid and solid as the pore width increases. However, the number of molecules adsorbed in the larger micropores increases with pore width and leads to the higher extent of adsorption [7]. We also observed the crossing in adsorption isotherms and this is due to the packing effect that leads to the difference in maximum density in each pore [46]. The high maximum density of 6.3 Å pore for CO_2_ and that of 7.5 Å pore for CH_4_ is due to the reason that fluid particles can tightly fit inside the pore as a single layer. For pores larger than 9 Å, molecules can form a monolayer along the pore wall and then the additional layers next to the monolayer. The monolayer and pore filling mechanism can be observed for the larger pore widths of 20 Å, as one can see the slope of isotherms increases gradually due to the formation of monolayer and then it shows a small jump in adsorption isotherm at high pressures due to the pore filling behavior. The additional capillary condensation can be observed in the case of adsorption in the larger mesopores.

The snapshots of CO_2_ molecules in the finite pores of 6.3, 9 and 20 Å widths at 273 K are shown in Figure 12 at various pressure values. In these figures, the black spheres represent carbon atoms of graphene layers, and for clarity we show only one graphene layer for each wall, while the big red and small blue spheres represent carbon and oxygen atoms of carbon dioxide molecule. The number of layers in the 6.3, 9 and 20 Å width is 1, 2 and 6, respectively; however, for the larger pores, the higher layers are not quite clear due to the fact that CO_2_ molecules can rotate in any direction. It is noted that the behavior in each pore is similar irrespective of its width, that is, the adsorbed phase starts at low pressure by forming the two contact layers adjacent to the two walls due to the stronger solid-fluid interaction near the pore wall (except the 6.3 Å width that has a single layer). When the pressure is increased, these contact layers are complete and the inner cores are then filled up. In the larger pores, we can observe the presence of meniscus; the shape of meniscus is cylindrical and becomes flat at pressures close to the vapor pressure. It is noted that a study of meniscus is not possible with the simulation of infinite pores [27].

### 3.3. Simulation Isotherm for Gas Adsorption in Finite Length Tube Bundle Model

#### 3.3.1. Effects of Tube Diameter on Adsorption Isotherm

**Figure 13:**
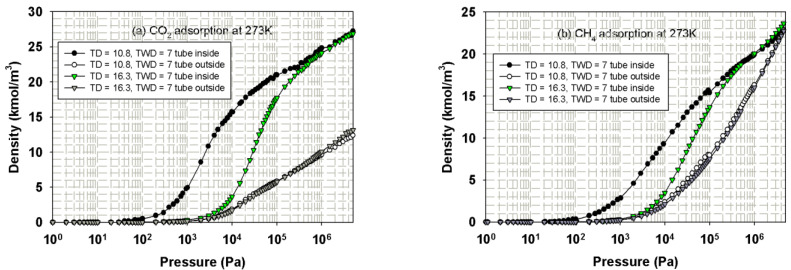
Isotherms of CO_2_ (**a**) and CH_4_ (**b**) in bundles of tube size 10.8 and 16.3 Ǻ at 273 K.

**Figure 14:**
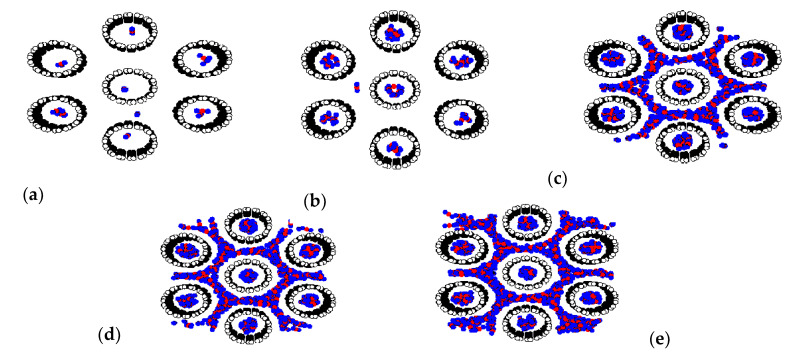
Snapshots of CO_2_ adsorption at various pressures: *p* = 0.3 (**a**), 1 (**b**), 900 (**c**), 8000 (**d**) and 90,000 Pa (**e**) in 10.8 Ǻ tubes at 273 K and TWD of 7 Å.

**Figure 15:**
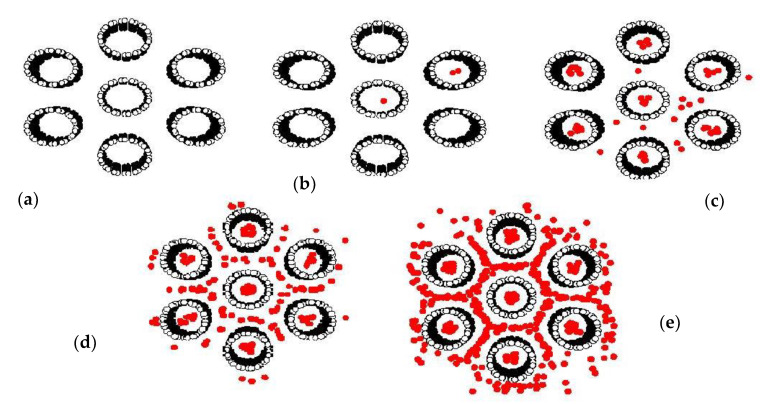
Snapshots of CH_4_ adsorption at various pressures: *p* = 0.3 (**a**), 1 (**b**), 900 (**c**), 8000 (**d**) and 90,000 Pa (**e**) in 10.8 Å tubes at 273 K and TWD of 7 Å.

The simulated isotherms versus pressure for CH_4_ and CO_2_ in bundle of SWCNs with a tube wall distance of 7 Ǻ at 273 K for 10.8 and 16.3 Ǻ tubes are shown in Figure 13. The snapshots of CO_2_ and CH_4_ in tube diameter of 10.8 Ǻ are shown in Figure 14 and Figure 15, respectively, to show the preferential adsorption at low loadings. First, we discuss the effect of tube diameter on CH_4_ and CO_2_ adsorption and then compare the adsorption mechanism between both adsorbates.

Adsorption isotherms of CH_4_ and CO_2_ in bundles of carbon nanotubes of different sizes are shown in Figure 13a,b, respectively; the similar behavior for the bundles of smallest tubes (10.8 Å) and those of larger tubes (16.3 Å) can be observed. At low pressures, fluid molecules are initially adsorbed in the interior of the tubes to form a monolayer because of the stronger solid-fluid potential inside the tube. The snapshots in Figure 14a,b for CO_2_ adsorption and Figure 15a–c for CH_4_ adsorption show this preferential adsorption inside the tube. As pressure is increased, adsorption continues to occur inside the tube and adsorption in the various interstices outside the tubes can be noticed, as shown in Figure 14c–e and Figure 15d,e. The initial adsorption pressure for the bundles of larger tubes is greater than that for those of smaller tubes. This is due to the stronger interaction between fluid and solid in the case of smaller tubes. The following features are also observed: (i) the adsorption density inside the tube increases gradually with pressure due to the molecular layering and the pore-filling mechanisms, and (ii) the density outside the tube also increases gradually. When pressure is high enough, the capillary condensation can be observed in interstices between the bundles. The adsorption isotherm outside the tube for larger and smaller tubes with narrow tube wall distance (TWD) are not much different; this is because the interstices between tubes can be packed with one layer as one can see from Figure 14c and Figure 15d for CO_2_ and CH_4_, respectively. The simulation isotherm outside the tube of CO_2_ is less than that of CH_4_ at high pressures. This may be due to the difference in molecular structure between CH_4_ and CO_2_; a non-spherical molecule of CO_2_ which is similar to the linear model of N_2_ is used in this study. It is reported in the literature that the non-spherical molecule of N_2_ can lie flat on the graphene wall, but it is difficult to occur in the multilayer [47]. Therefore, the molecular shape is important in the explanation of adsorption in confined space with curvature This is because it may affect the structure of adsorbed phase and packing density.

We now turn to the discussion of adsorption behavior between CH_4_ and CO_2_ in bundles of tubes. CO_2_ molecules initially adsorb at lower pressures than those of CH_4_ for the same tube diameter. At the same pressure and tube diameter, adsorption density inside the tube for CO_2_ is greater than that for CH_4_, which is similar to those observed in the experimental data and simulation results for the slit pore model. This is due to the quadrupole effect in the case of CO_2_, which leads to the stronger fluid-solid interaction as discussed earlier.

#### 3.3.2. Effects of Tube Wall Distance

**Figure 16:**
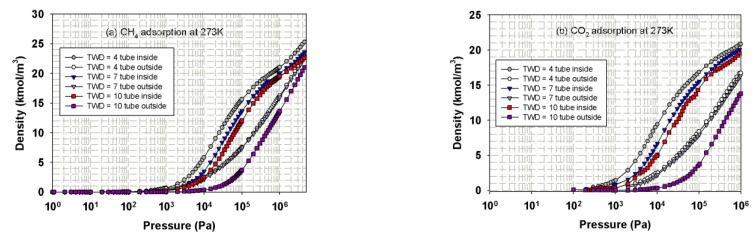
Adsorption isotherms of CH_4_ (**a**) and CO_2_ (**b**) in bundles of 16.3 Å tubes at 273 K at various tube wall distance of 4, 7 and 10 Å.

Having seen the effects of the tube diameter, we then discuss the effects of tube wall distance on adsorption mechanism. The simulation isotherms of CH_4_ and CO_2_ in bundles of SWCNs of 16.3 Ǻ at 273 K at various tube wall distances of 4, 7 and 10 Ǻ are shown in Figure 16.

The individual adsorption isotherms for adsorption inside the tube (the top curves as shown in pore) and outside the tube (bottom curves as shown in bulk) for tube bundle of SWCN having diameter of 16.3 Å with different tube wall distances are presented in Figure 16, and the following observations can be noted. At low pressures, fluid particles are initially adsorbed inside the tubes if the separation spacing is less than 10 Å due to the stronger solid-fluid potential inside the tube as discussed in Section 3.3.1. For larger tube wall distance, the behavior is different from the TWD less than 10 Å tubes in that, at low loadings, adsorption occurs both inside the tubes and in the cusp interstices where the solid-fluid potential is strong enough to compete with that inside the tube. This is the direct result from the difference in size of the cusp interstices and the tube diameter. However, when the tube radius is greater than the separation spacing between tubes, adsorption in the cusp interstices may occur first followed by that in the tube interior [28]. In the case of CO_2_ adsorption outside the tubes of 16.3 Å diameter and TWD of 10 Å, the isotherm increases gradually due to the layering outside the tube walls and then steeply changes in adsorption isotherm can be observed. This may be due to the capillary condensation behavior of CO_2_ molecules in the cusp interstices at sufficiently high pressure.

### 3.4. Pore Size Distribution (PSD) Determination

**Figure 17:**
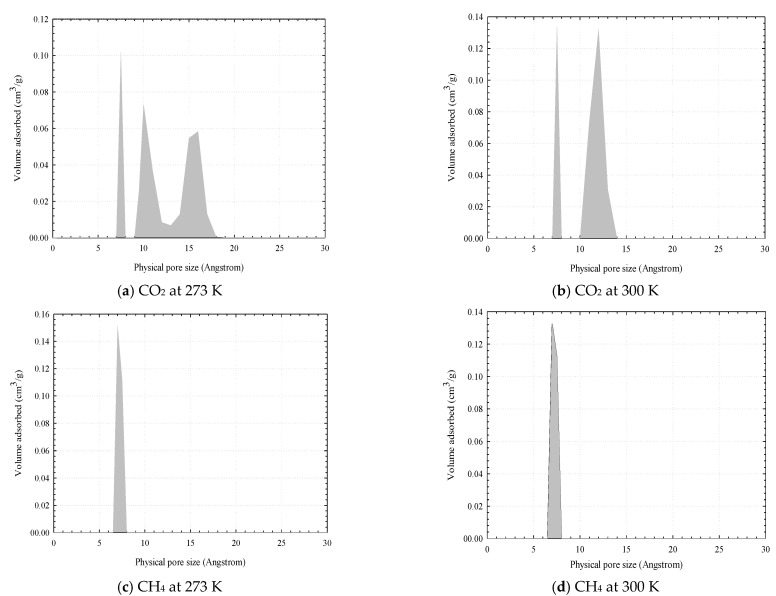
Pore size distribution obtained for LAC1 using the isotherms of CO_2_ at 273 K (**a**), CO_2_ at 300 K (**b**), CH_4_ at 273 K (**c**) and CH_4_ at 300 K (**d**).

A method to determine the PSD developed based on GCMC simulations and measured isotherm data of CO_2_ proposed by Samios et al. [48] is used in this study. The set of simulation results are compared against the corresponding experimental isotherm counterpart by using an optimization function of MATLAB code. The PSD obtained by using adsorption isotherms of CO_2_ and CH_4_ in activated carbons at 273 and 300 K are shown in Figure 17. The total pore volume of LAC1 obtained by CO_2_ adsorptions at 273 and 300 K are 0.3921 and 0.3707 cm^3^/g, respectively, and those obtained by CH_4_ adsorptions at 273 and 300 K are 0.2633 and 0.2450 cm^3^/g, respectively. These values differ from those obtained from nitrogen isotherms at 77 K determined by a density functional theory (DFT) method. The total pore volume determined from DFT is 0.27 cm^3^/g for the longan seed activated carbon. The differences between these results may be due to that the 3-center-LJ model of CO_2_ does not completely fill in the larger pores because of its orientation requirement and the experiment is operated at pressures lower than its vapour pressure. The total pore volume determined from our model is greater than that obtained from the DFT method and experimental data for N_2_. This is because the kinetic energy of CO_2_ is greater than that of N_2_ (because of the higher adsorption temperature). Thus, the diffusion rate of CO_2_ into the narrow micropores is faster than that of N_2_, which agreed with the experimental data that the isotherm for CO_2_ adsorption in the T3A carbon at 273 K was easily obtained than that for N_2_ at 77 K [49]. The total pore volume obtained from methane adsorption isotherms is less than those obtained from the adsorption of N_2_ and CO_2_; again this because methane cannot fill completely the larger pores at the operating temperatures. This leads to the decrease of total pore volume with an increase temperature.

**Figure 18:**
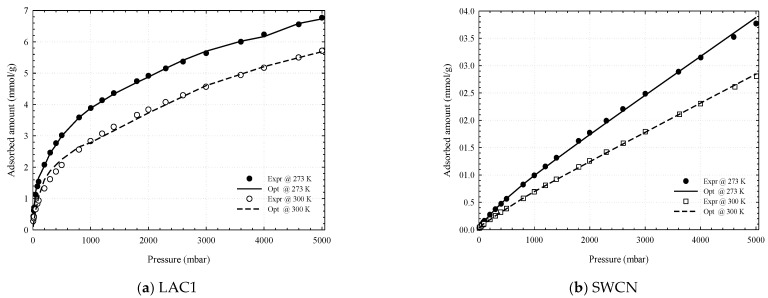
Comparison between the experimental data and reconstructed adsorption isotherms obtained from PSD determination for LAC1 (**a**) and SWCN (**b**) using the adsorption isotherms of CO_2_ at 273 and 300 K.

The reconstructed adsorption isotherm obtained from the PSD of CO_2_ data at 273 and 300 K agrees well with the experimental results for activated carbon and carbon nanotubes, as shown in Figure 18. Therefore, the CO_2_ data at low pressures can be used to determine the PSD for the micropores, while the N_2_ adsorption isotherm is used to determine the total pore volume in porous carbons. For better accuracy, the PSD obtained from nitrogen isotherm should be used together with the PSD obtained from CO_2_ isotherm.

**Figure 19:**
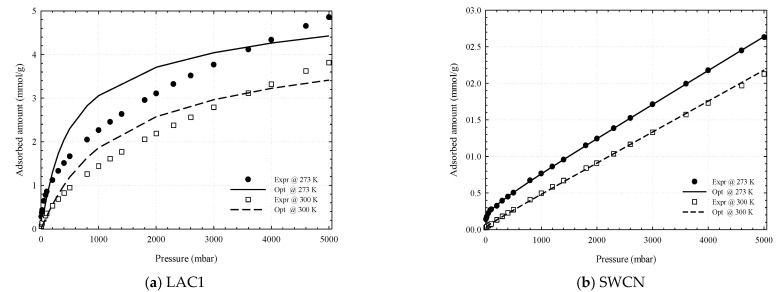
Comparison between the experimental data and reconstructed adsorption isotherms obtained from PSD determination for LAC1 (**a**) and SWCN (**b**) using the adsorption isotherms of CH_4_ (**b**) at 273 and 300 K.

Having seen the PSD obtained from the CO_2_ adsorption isotherm, we now turn to the PSD obtained from that of CH_4_ at 273 and 300 K as shown in Figure 17. The micropore volume using the GCMC for LAC1 is 0.34 cm^3^/g while SWCN composed of pore having diameter of 10.8 Ǻ with TWD 4 Ǻ is observed. The reconstructed isotherms from PSD function agree well with the experimental data for SWCN as shown in Figure 19, but it cannot be described well for the activated carbon. This may be due to the supercritical fluid condition of methane at these temperatures and then the fluid condensation inside the pore cannot be observed. The different probe and temperature can affect the evaluation of PSD of porous carbons. It is noted that the small pores of width less than 10 Ǻ can be observed in the case of using GCMC simulation; however, they are not found for PSD obtained from the DFT calculation [46]. Although the simulation isotherm obtained for finite-length slit pore model proposed in this study cannot present the entire structure range of porous carbon, indeed some important characteristics are revealed.

### 3.5. Prediction Adsorption Isotherm of CO_2_/CH_4_ Mixture

**Figure 20:**
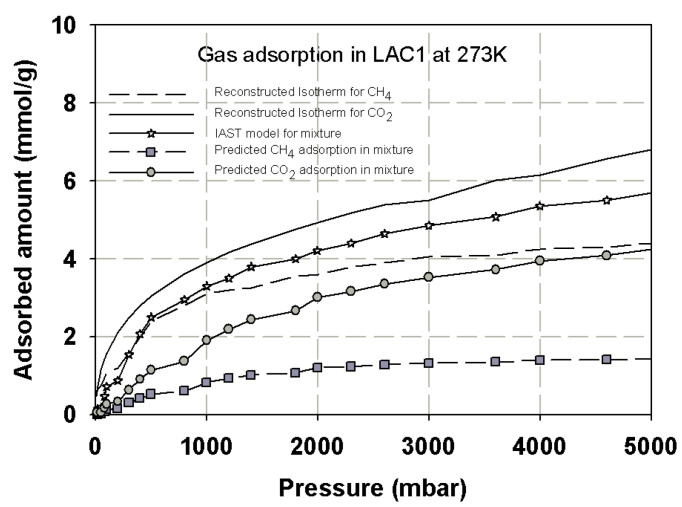
Simulation isotherms obtained for CH_4_ and CO_2_ for binary mixture of equimolar CO_2_/CH_4_ in slit pore model at 273 K.

In this section, the reconstructed adsorption isotherm obtained for single component of CO_2_ and CH_4_ in finite length slit pore derived in the previous section is used to predict the adsorption isotherm of their mixture. The adsorption equilibrium for multicomponent system equation based on thermodynamic theory is used for analysis of the adsorbed phase and gas phase components and for describing the adsorption behavior of the mixture. It is reported in the literature that the error between the adsorption isotherms obtained from the Vacancy Solution Theory (VSM) and the experimental data for binary mixture is about 5% [36]. Therefore, in this study we used this equation and Ideal Adsorption Solution Theory (IAST) [8] to evaluate the adsorption component of each gas in adsorbed phase, and the results are shown in Figure 20. The simulation isotherm obtained for CO_2_/CH_4_ in finite-length slit pores having widths of 7.5 and 13.5 Ǻ was also investigated by the GCMC simulation as shown in Figure 20. The adsorption isotherm of gas mixture is less than the adsorption isotherm of CO_2_ but it is greater than that of CH_4_, which is similar to that observed in the experimental section. The mole fraction of CO_2_ adsorption in activated carbon is about 0.65–0.75, while that of CH_4_ is around 0.25–0.35. Therefore, the selectivity of CO_2_–CH_4_ at equimolar feed is 1.8–3.0, which is close to that observed in the experimental study of Kurniawan et al. [50]. The simple model of finite length slit pore can be used to described the adsorption isotherm of single component and binary mixture quite well.

## 4. Conclusions

In this study the finite-length pore models of slit shaped pore and tube bundle were used to investigate the adsorption mechanism of CO_2_, CH_4_ and their mixture in activated carbons and carbon nanotubes, respectively. The pore size distribution obtained for pure component study of each gas using the Monte Carlo simulation agrees well with that obtained using nitrogen adsorption isotherm determined by the DFT; however, the PSD obtained from MC shows a pore size smaller than 10 Ǻ, which was not observed from the DFT. The PSD obtained by the MC and finite-length pore model were used to predict the adsorption isotherm for binary mixture and compare it with the experimental results, and the agreement is satisfactory.

## Figures and Tables

**Table 1:** Molecular parameters for methane and nitrogen (r_NN_ is the separation distance between two N atoms).

Fluid	$\sigma_{ff},\;Å$	$\varepsilon_{ff}/k,\;K$	$r_{NN},\;Å$
CH_4_	3.730	148.0	-
N_2_ (2 LJ sites)	3.320	36.4	1.1

**Table 2:** Molecular parameters for carbon dioxide.

Parameter	Value	Parameter	Value
_σ_^c-c^	2.757 Å	_ε_^c-c^/k	28.129 K
_σ_^o-o^	3.033 Å	_ε_^o-o^/k	80.507 K
_q_^c^	0.6512e	_q_^o^	−0.3256e
_λ_^c-o^	1.149 Å		

**Table 3:** Porous properties of carbons used in this study.

Sample	BET Surface Area (m^2^/g)	Micropore Volume (cm^3^/g)	Meso- and Macropores Volume (cm^3^/g)	Total Pore Volume (cm^3^/g)
SWCN	853	0.18	1.11	1.29
LAC1	538	0.20	0.07	0.27
LAC2	705	0.26	0.11	0.37

## Data Availability

Not applicable.

## Sample Availability

Samples of the compounds are not available from the authors.
